# Tunable plasmon lensing in graphene-based structure exhibiting negative refraction

**DOI:** 10.1038/srep41788

**Published:** 2017-02-02

**Authors:** Shifeng Zhong, Yanxin Lu, Chao Li, Haixia Xu, Fenghua Shi, Yihang Chen

**Affiliations:** 1 Guangdong Provincial Key Laboratory of Quantum Engineering and Quantum Materials, School of Physics and Telecommunication Engineering, South China Normal University, Guangzhou 510006, China; 2 School of Information Science and Technology, Zhongkai University of Agriculture and Engineering, Guangzhou 510225, China

## Abstract

We propose a novel method to achieve tunable plasmon focusing in graphene/photonic-crystal hybrid structure exhibiting all-angle negative refraction at terahertz frequencies. A two-dimensional photonic crystal composed of a square lattice of dielectric rods is constructed on the substrate of a graphene sheet to provide the hyperbolic dispersion relations of the graphene plasmon, giving rise to the all-angle plasmonic negative refraction. Plasmon lensing induced from the negative refraction is observed. We show that the ultracompact graphene-based system can produce sub-diffraction-limited images with the resolution significant smaller than the wavelength of the incident terahertz wave. Moreover, by adjusting the Fermi energy of the graphene, the imaging performance of the proposed system can remain almost invariant for different frequencies. Our results may find applications in diverse fields such as subwavelength spatial light manipulation, biological imaging, and so forth.

As the rapid development of information processing in the past few decades, miniaturization and integration have become the prevailing trend of electronic-photonic system used for digital communication. Suffered from the diffraction limit, traditional photonic devices are bulky and cannot be integrated with the electronic components on the same chip. Surface plasmon-based photonics offer a solution to this dilemma. Surface plasmons (SPs) are electromagnetic wave which propagates along the metal/dielectric interface and can be guided by metallic nanostructures beyond the diffraction limit^1^,^2^. Perpendicular to the interface, SPs have subwavelength-scale confinement^3^. This remarkable capability has unique prospects for the design of nanoresolution optical imaging techniques^4^,^5^, filters^6^, sensors^7^,^8^, highly integrated all-optical diode^9^, etc. Recently, negative refraction of SPs in metallic nanostructures has attracted great attention because it is the foundation of a variety of new electromagnetic effects and applications, such as superlensing^10^,^11^, negative Doppler effect, as well as novel guiding, localization and nonlinear phenomena^12^,^13^. Plasmonic superlens based on the negative refraction can enhance the near-field source components and form high-resolution sub-diffraction-limit images^5^. However, previous plasmonic superlens does not work at terahertz (THz) range, their focusing performance are generally difficult to be tuned.

Graphene, a two-dimensional material where carbon atoms are arranged in a honeycomb lattice, has attracted significant attention due to its linear dispersion and zero electron density of states at the Dirac point^14^,^15^. It was demonstrated that plasmons can be excited on a graphene layer^16^. Compare to the surface plasmons in metal, graphene plasmons exhibit higher confinement and relatively low propagating loss^17^. Recently, graphene plasmons have been successfully excited and observed in experiments^18^,^19^. Graphene-based plasmonic devices such as waveguide^20^, sensor^21^, directionalcoupler^22^, modulaters^23^,^24^, absorbers^25^, oscillators^26^, were proposed theoretically or experimentally. Moreover, metasurface was demonstrated that it can provide a versatile platform for manipulating the propagation of graphene plasmons. It was reported that plasmon waveguide^27^, plasmon Luneburg lens^27^, and plasmon convex lens^28^ can be achieved by precisely designing the spatially inhomogeneous conductivity distributions across a graphene sheet. Geometrically tailored antennas were used to launch and focus the infrared graphene plasmons^29^. Anisotropic uniaxial metasurfaces were design to realize topological transistions^30^ and anisotropic propagation of the graphene plasmons^31^. In contrast to the metal-based plasmonic devices, the performance of the graphene-based plasmonic structures can be efficiently adjusted by chemical doping or electrical gating at mid-infrared and THz frequencies.

In this paper, we show that all-angle negative refraction of plasmons can be achieved in graphene/photonic-crystal hybrid structure. We demonstrate that plasmon focusing based on the negative refraction can be used to attain subdiffraction-resolution imaging at THz frequencies. Our results show that the resolution of the image is significant smaller than the wavelength of the graphene plasmon. Furthermore, the focusing performance of the proposed structure can be maintained for different frequencies by changing the Fermi energy of the graphene.

## Results and Discussions

**Figure 1:**
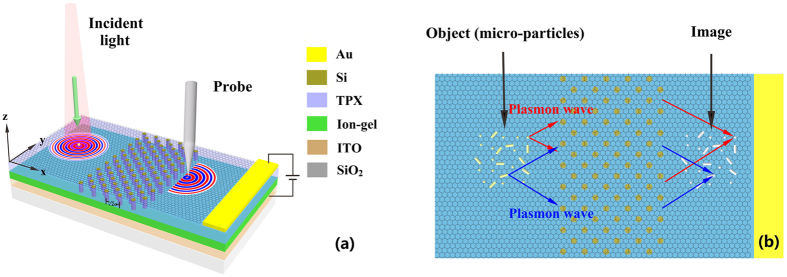
Schematic of the graphene/photonic-crystal hybrid system. (**a**) Graphene plasmon is excited by illuminating the metal micro-particle and is focused by the graphene/photonic-crystal structure. A probe of a near-field optical microscopy is used to detect the field information of the focused plasmon. (**b**) Image of the sample (metal micro-particles) can be obtained by scanning the imaging region with the probe.

Figure 1(a) shows the schematic diagram of the considered graphene-based system, where a graphene sheet is deposited on the polymethylpentene (TPX)^32^ substrate patterned with a two-dimensional photonic crystal (2D PC). The PC is composed of a square array of Si pillars with lattice constant *a* and pillar radius *r*. The surface normal of the PC is along *ΓM* [(11) direction]. Ion-gel is sandwiched between TPX and ITO/SiO_2_ substrate. Gold patch on the graphene sheet is used as an electrode and ITO is used as the other electrode. In the following simulations, graphene sheet is treated as an ultrathin layer with thickness 0.5 nm, the radius of Si pillars is set as *r* = 0.3*a*, where *a* = 2 μm. Metal micro-particle, illuminated by an incident beam, can work as an optical antenna which can convert the incident light into a localized near-field. When such an antenna is placed on graphene, the high-momentum near-field components match the wave vector of the graphene plasmon, thus exciting graphene plasmons^29^, as shown in Fig. 1(a). An image of the micro-particle forms on the opposite side of the graphene-PC region through the plasmon lensing effect. A probe of a near-field optical microscopy is used to detect the field information. The optically induced field information image of metal micro-particles can be obtained by scanning the imaging region with the probe, as shown in Fig. 1(b).

Before the investigation of the properties of the propagating graphene plasmons, we need to evaluate the effective index of the graphene plasmons. The complex surface conductivity of graphene can be deduced from Kubo formula^33^. In the terahertz frequencies, the optical conductivity contribution of the interband transition can be neglected^34^. Under the room temperature (*T* = 300 *K*), the graphene optical conductivity obeys a Drude dispersion model^35^


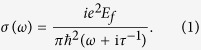


In the following simulations, the relaxation time is set as τ = 6.4 × 10^−13^ s, which corresponds to the measurement under the DC mobility *μ* = 10000 cm^2^/(V·s)^36^,^37^. Considered the graphene sheet is surrounded with dielectrics of constants *ε*_*r*1_ and *ε*_*r*2_, the dispersion relation of plasmon in graphene can be written as^37^


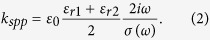


The effective index of the graphene plasmon can be obtained from *n*_*eff*_ = *k*_*spp*_/*k*_0_.

**Figure 2:**
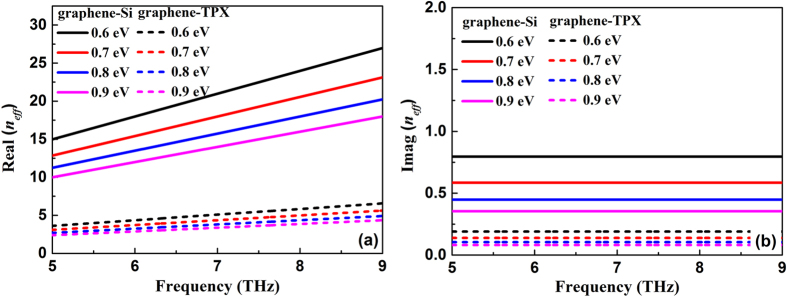
(**a**) Real and (**b**) imaginary parts of the effective index of graphene plasmon mode at graphene-silicon and graphene-TPX waveguides as a function of frequency under different Fermi energy levels.

Figure 2 shows the effective index of the graphene plasmon mode supported by a graphene sheet on silicon or TPX substrate for different Fermi energy levels. It can be seen that the real part of the effective index of the graphene plasmon mode decreases with the increase of the Fermi energy, which can be utilized in the tunable plasmonic devices. Moreover, as shown in Fig. 2(a), the real part of the effective index of the plasmon mode on the graphene-silicon waveguide is always much larger than that on the graphene-TPX waveguide. Such a high refractive index contrast will benefit for the construction of a PC. By forming a 2D PC in the substrate of the graphene layer, as shown in Fig. 1, one can manipulate the propagation of the graphene plasmon by means of the plasmonic band structures. It is seen from Fig. 2(b) that the imaginary part of the effective index of the graphene plasmon mode decreases as the Fermi energy increases, which indicates the propagation loss is smaller at higher Fermi energy because the propagation length is determined by
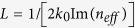
. In addition, the imaginary part of the plasmon effective index is almost independent of the frequency, as shown in Fig. 2(b).

**Figure 3:**
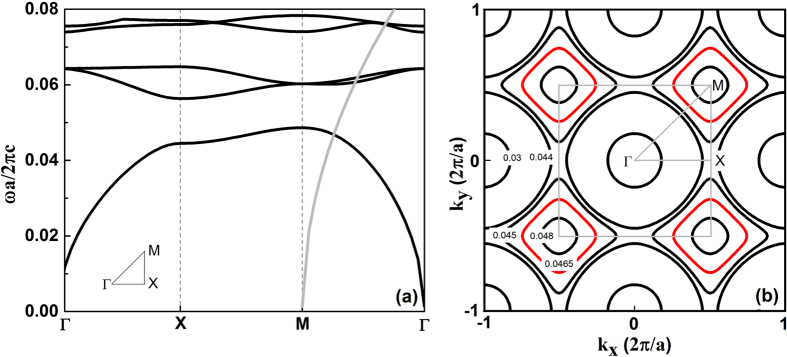
(**a**) Plasmonic band structures (the light line shifted to *M* is shown in grey) and (**b**) equifrequency contours of the first band for the considered graphene/PC hybrid structure where the Fermi energy of graphene *E*_*f*_ = 0.8 eV.

**Figure 4:**
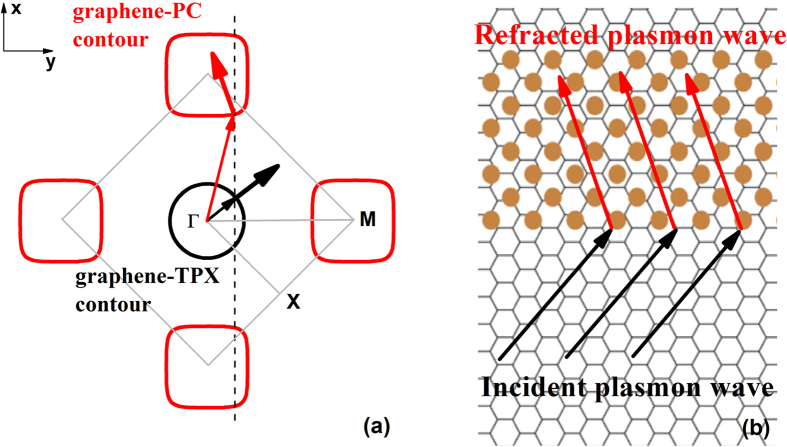
(**a**) Equifrequency contours of the plasmon modes in the graphene/PC hybrid structure at frequency *ω* = 0.0465 × 2π*c/a*. The Fermi energy of graphene *E*_*f*_* = *0.8 eV. Thick and thin arrows respectively indicate the directions of group-velocity and phase-velocity. (**b**) Diagram of the negative refraction behavior in the actual graphene-based system.

Next, we investigate the band structures of the graphene plasmon in the considered system. The eigenfunction of the graphene/PC hybrid structure was derived (see Methods). For the considered system at the Fermi energy *E*_*f*_ = 0.8 eV, we calculated the plasmonic band structures using the plane wave expansion method^38^, as shown in Fig. 3(a). It is seen that the first energy band has an intersection with the light line at frequency *ω* = 0.0468 × 2π*c/a*. Figure 3(b) illustrates the equifrequency contours of the first band of the graphene/PC hybrid system. It can be seen that the frequency contours are convex in the vicinity of *M*. In these cases, group velocity determined by ∇_*k*_*ω(k*) points toward *M*^38^. Therefore, for frequencies that correspond to all-convex contours, negative refraction will be occurred along the *ΓM* direction as illustrated in Fig. 4.

When the propagating plasmon is incident from the graphene-TPX region to the graphene-PC region, the wave-vector component parallel to the interface is conserved so that the boundary condition is satisfied^39^. For the considered system where the surface of the PC is normal to *ΓM* direction, if the equifrequency contour is convex, the incident plasmon wave will couple to a Bloch mode that propagates into the graphene-PC region on the negative side of the boundary normal direction. As shown in Fig. 4(a), the wave-vector of the Bloch wave refracts positively, while the energy flow undergoes a negative refraction. Hence negative refraction of the plasmon wave can be achieved in the first band. During the first band, ***k*** · **∇**_*k*_*ω(**k***) ≥ 0, it means that the group velocity is not opposite to the phase velocity along the *ΓM* direction. Therefore, the negative refraction of the plasmon wave in the considered graphene-based system does not involve a negative effective index.

**Figure 5:**
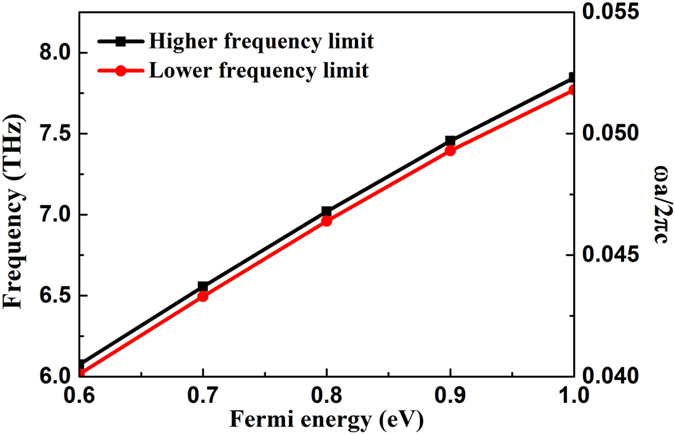
AANR frequency range as a function of the Fermi energy of graphene. Black and red lines, respectively, represent the higher- and lower-frequency limits for the AANR of the graphene plasmon.

Such a negative refraction phenomenon can be used to realize plasmonic imaging with subwavelength resolution. To achieve better imaging performance, it requires that the graphene plasmon wave at any incident angle experiences negative refraction when entering the graphene-PC region. To achieve this phenomenon, the graphene-PC contour should be convex and larger than the graphene-TPX contour. In this case, the incident wave at any incident angle, from −90° to 90°, can be refracted with negative refraction angle in the graphene-PC region, which is named the all-angle negative refraction (AANR). From the calculation results, we find that AANR can occur in the first band. When the Fermi energy is set as 0.8 eV, the AANR frequency range is from 0.0464 × 2π*c/a* to 0.0468 × 2π*c/a*, i.e., from 6.96 to 7.02 THz. Compared to the previous design of AANR using PC structure^40^, the AANR frequency range of the graphene-based system is narrow. This can be understood that the large and strongly frequency-dependent effective indices of the graphene plasmons, as shown in Fig. 2, make the plasmonic band structures of the graphene-PC system flattened, resulting in the greatly compressed AANR band. By varying the Fermi energy of graphene, the AANR frequency range can be tuned. Figure 5 shows the dependence of the higher- and lower-frequency limits for the AANR on the Fermi energy. One can see that the AANR frequency range can be tuned effectively from around 6.02 THz to around 7.85 THz by increasing the Fermi energy from 0.6 to 1.0 eV. Such a tunable AANR of graphene plasmon is essential for tunable THz superlensing.

**Figure 6:**
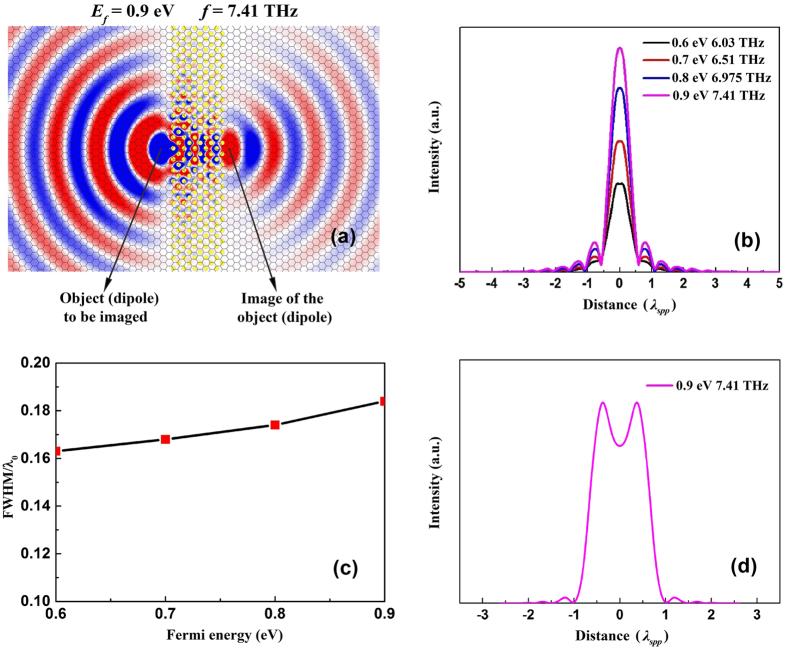
(**a**) *E*_z_ field of a plasmon point source (dipole) and its image across a graphene-PC region in case of *E*_*f*_ = 0.9 eV and *f* = 7.41 THz. (**b**) Electric field intensities at the image plane and (**c**) FWHM of the images corresponding to the cases of *E*_*f*_ = 0.6 eV and *f* = 6.03 THz, *E*_*f*_ = 0.7 eV and *f* = 6.51 THz, *E*_*f*_ = 0.8 eV and *f* = 6.975 THz, *E*_*f*_ = 0.9 eV and *f* = 7.41 THz, respectively. (**d**) Electric field intensity at the image plane for two point sources (dipoles) separated by 
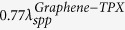
 apart.

To demonstrate how the AANR of the graphene plasmon can be used for THz imaging, we have performed finite-difference time-domain (FDTD) simulations with perfectly matched layer boundary conditions on the graphene-based hybrid system. The simulations were conducted via FDTD SOLUTIONS, a commercial 3D FDTD-method Maxwell solver. As shown in Fig. 1(a), the illuminated metal particle acts as a resonant dipole antenna which launches graphene plasmon. Here, a dipole is placed at a distance 0.35*a* from the left-hand surface of the PC. Figure 6(a) depicts a snapshot of *E*_*z*_ field in the considered system with *E*_*f*_ = 0.9 eV. Here the frequency is 0.0494 × *c/a* = 7.41 THz, which lies within the AANR frequency range. It is seen that the plasmon wave is focused on the right-hand side of the graphene-PC region. The central position of the image of the dipole is at a distance 1.20*a* from the right-hand surface of the PC. Similar plasmon imaging behavior can be achieved at different frequencies by choosing a suitable Fermi energy of graphene.

To evaluate the imaging quality, Fig. 6(b) plots the electric field intensity at the image plane in cases of *E*_*f*_ = 0.6 eV and *f* = 6.03 THz, *E*_*f*_ = 0.7 eV and *f* = 6.51 THz, *E*_*f*_ = 0.8 eV and *f* = 6.975 THz, *E*_*f*_ = 0.9 eV and *f* = 7.41 THz, respectively. It can be seen from Fig. 6(b) that the full width half maximum (FWHM) of the images changes only a little as the Fermi energy varies. To elucidate this issue, we calculated the effective parameters of the graphene plasmons and showed the results in Table 1. It can be seen that the wavelengths of the graphene plasmons in the input and output graphene-TPX waveguides are almost the same for the four cases. Moreover, the ratios of the real part of 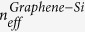 to that of 
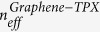
 are also very close although both 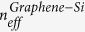 and 
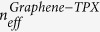
 decrease as the frequency and Fermi energy increase. This means that the Bragg scattering effects of the plasmon waves inside the graphene-PC region are similar for the four cases, resulting in their similar imaging performance. It can also be seen from Fig. 6(b) that the image intensity increases obviously as the Fermi energy increases. This is due to the decrease of the propagation loss with the increasing Fermi energy of graphene. It is shown in Table 1 that, as the Fermi energy increases, the imaginary parts of the effective indices of the graphene plasmons decrease, and therefore the propagation loss of the plasmon wave decreases as well.

For the comparison of the imaging resolution at different Fermi energy, we plot the FWHM of the point images versus the Fermi energy in Fig. 6(c). It is seen that the FWHM value changes from 0.163λ_0_ to 0.187λ_0_ (λ_0_ is the wavelength of terahertz wave in vacuum) as the Fermi energy increases from 0.6 eV to 0.9 eV, which means that tunable imaging has been achieved at THz frequency range. Our simulations show that such a system can resolve two point sources, oscillating in phase, with a distance of 
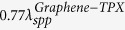
 between them. The proposed graphene-based system provides a new way for controlling the propagations of plasmon wave at deep subwavelength scale.

## Conclusions

In summary, we reported a novel design of graphene/PC hybrid structure to achieve tunable plasmon lensing. We showed that a 2D PC patterned on the substrate of a graphene sheet can realize all-angle negative refraction of the graphene plasmon in the first energy band, which results from the hyperbolic dispersion relations of the graphene/PC structure. We also showed that such a negative refraction of graphene plasmon can be electrically tuned by adjusting the Fermi energy of graphene, making adjustable wide-range negative refraction possible. In the presence of the tunable negative refraction effect, subdiffraction imaging was attained and the imaging performance can be maintained for different frequencies by changing the Fermi energy of the graphene. Our results suggest an effective mechanism for controlling the propagation of graphene plasmon and may find applications in ultra-compact subwavelength spatial light modulators.

## Methods

### Derivation of the dispersion relation for plasmons in graphene/PC hybrid structure

Consider a graphene sheet is deposited on a 2D PC, which is composed of a 2D array of Si cylinders surrounded by TPX background. Assume that the radius and the dielectric constant of Si cylinders is *r* and *ε*_1_ respectively, the Si cylinders are arranged in a square with a lattice constant of *a*, the dielectric constant of TPX is *ε*_2_. The graphene-Si region and the graphene-TPX region have different values of effective index for the in-plane plasmons.

In the THz frequency range, the optical conductivity of graphene obeys the Drude dispersion model (i.e. Eq. (1) in the main text). The effective index of the graphene plasmon can be written as *n*_*eff*_ = *k*_*spp*_/*k*_0_ = *k*_*spp*_*c/ω*, where the wave vector of the graphene plasmon *k*_*spp*_ can be described by Eq. (2). By combining Eqs (1) and (2), we can obtain the effective dielectric constant


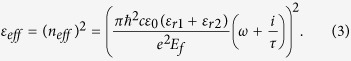


If we neglect the imaginary part of the effective dielectric constant, Eq. (3) can be simplified as


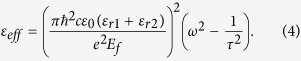


Therefore, the effective dielectric constant for the graphene-Si and graphene-TPX regions can be written as


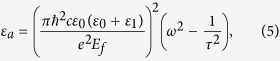



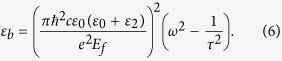


Assume that the graphene plasmon propagates along the *x* direction, the three field components can be expressed as


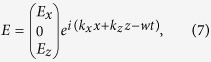



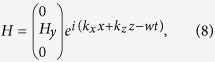


where *k*_*x*_ = *k*_*spp*_, 
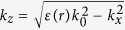
, *ε(r*) is the dielectric constant of Si or TPX. Since the energy of the plasmon is strongly confined at the graphene sheet, we only consider the *E*_*z*_ components


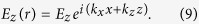


For the propagating plasmon (TM mode), *k*_*spp*_ can be expressed by valid Helmholtz equation


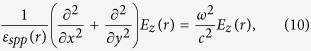


where *ε*_*spp*_(*r*) is the effective dielectric constant for the graphene-Si or graphene-TPX region. By expanding *E*_*z*_(*r*) and *ε*_*spp*_(*r*) in reciprocal space, the eigenfunction of our system can be derived as


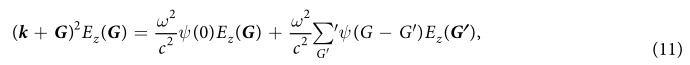


where the *ψ(G*) and *E*_*z*_(*G*) are the Fourier expansion coefficient of *ε*_*spp*_(*r*) and *E*_*z*_(*r*). *ψ(G*) can be expressed as,


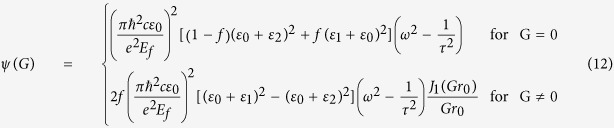


Combining Eqs (11) and (12), the eigenfunction can be obtained as


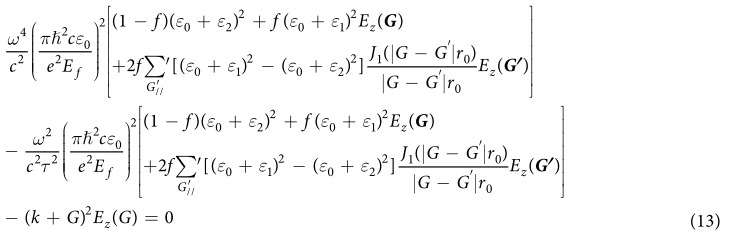


To facilitate the calculation, we define three matrices A, B, and C:


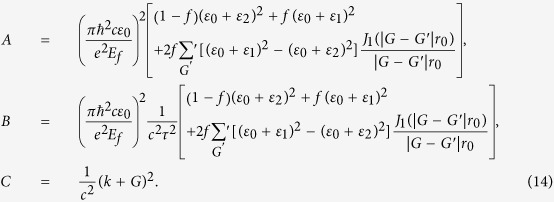


Combining Eqs (13) and (14), the eigenfunction can be simplified as,


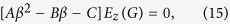


where *β *= *ω*^2^/*c*^2^. By changing the form of the Eq. (15), we obtain the expression


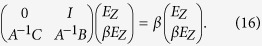


By using Eq. (16), we can calculate the dispersion relation of plasmons in graphene/PC structure.

### Fabrication process of the proposed graphene-based structure

**Figure 7:**
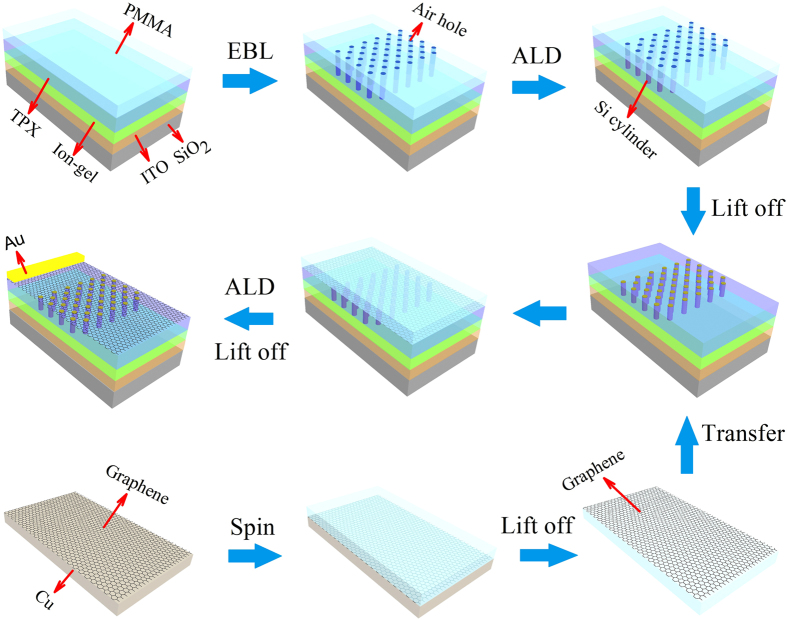
Schematic of the fabrication process for the proposed graphene-based structure.

Scheme for fabricating our proposed device is shown in Fig. 7. First, an ITO-coated silica substrate is spin coated, in turn, with the ion-gel, TPX and PMMA layers^41^. Next, the air holes in the PMMA and TPX film are fabricated by electron beam lithography (EBL)^42^. Then, the amorphous Si is deposited and formed the Si cylinder with the depth just right in the TPX film using the atomic layer deposition (ALD) method^43^. After these, the PMMA film is lifted off using the acetone so that the photonic crystal can be formed inside the TPX layer.

On the other hand, a monolayer graphene is fabricated on Cu substrate using chemical vapour deposition (CVD) method^44^. Then, a PMMA film is spin coated on the graphene, followed by a lifting-off process to obtain the monolayer graphene on the PMMA substrate (graphene-PMMA)^45^. Next, the graphene-PMMA structure is transferred onto the pre-fabricated photonic-crystal-patterned TPX layer. Finally, the PMMA film is lifted off using the acetone, and the gold electrode is deposited on the graphene using the ALD method.

## Additional Information

**How to cite this article**: Zhong, S. *et al*. Tunable plasmon lensing in graphene-based structure exhibiting negative refraction. *Sci. Rep.* **7**, 41788; doi: 10.1038/srep41788 (2017).

**Publisher's note:** Springer Nature remains neutral with regard to jurisdictional claims in published maps and institutional affiliations.

## Figures and Tables

**Table 1:** Effective parameters of the graphene plasmons corresponding to Fig. 6.

*E*_*f*_ (eV)	*f* (THz)	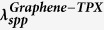 (10^−5^ m)	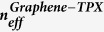	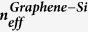
0.6	6.03	1.1151	4.464 + 0.184*i*	18.170 + 0.749*i*
0.7	6.51	1.1162	4.131 + 0.158*i*	16.814 + 0.642*i*
0.8	6.975	1.1112	3.873 + 0.134*i*	15.763 + 0.562*i*
0.9	7.41	1.1077	3.657 + 0.123*i*	14.886 + 0.500*i*
